# Modeling the future of irrigation: A parametric description of pressure compensating drip irrigation emitter performance

**DOI:** 10.1371/journal.pone.0175241

**Published:** 2017-04-06

**Authors:** Pulkit Shamshery, Ruo-Qian Wang, Davis V. Tran, Amos G. Winter V

**Affiliations:** Global Engineering and Research Laboratory, Department of Mechanical Engineering, Massachusetts Institute of Technology, Cambridge, MA, United States of America; University of Washington, UNITED STATES

## Abstract

Drip irrigation is a means of distributing the exact amount of water a plant needs by dripping water directly onto the root zone. It can produce up to 90% more crops than rain-fed irrigation, and reduce water consumption by 70% compared to conventional flood irrigation. Drip irrigation may enable millions of poor farmers to rise out of poverty by growing more and higher value crops, while not contributing to overconsumption of water. Achieving this impact will require broadening the engineering knowledge required to design new, low-cost, low-power drip irrigation technology, particularly for poor, off-grid communities in developing countries. For more than 50 years, pressure compensating (PC) drip emitters—which can maintain a constant flow rate under variations in pressure, to ensure uniform water distribution on a field—have been designed and optimized empirically. This study presents a parametric model that describes the fluid and solid mechanics that govern the behavior of a common PC emitter architecture, which uses a flexible diaphragm to limit flow. The model was validated by testing nine prototypes with geometric variations, all of which matched predicted performance to within R^2^ = 0.85. This parametric model will enable irrigation engineers to design new drip emitters with attributes that improve performance and lower cost, which will promote the use of drip irrigation throughout the world.

## Introduction

The objective of this work was to analyze the coupled fluid-structure interaction within a pressure compensating (PC) drip irrigation emitter. The term ‘pressure compensating’ refers to drip emitters that maintain a constant flow rate independent of the applied pressure. This attribute is valuable for maintaining uniform water flow distribution throughout a farm field. The minimum pressure required to induce the specified flow rate is called the ‘activation pressure’. The primary contribution of this paper is a parametric model that enables PC drip emitters to be designed with a desired flow rate and activation pressure given their internal features and geometry. The robustness of this analytical model to changes in individual geometric parameters in the emitter was tested by using eight different emitter configurations, which were based on the design of a commercially-available, 8 L/hr pressure compensating emitter.

Promoting the use of drip irrigation is important because, compared to rain-fed and flood irrigation, it can increase yields by 20-90% depending on crop type, grow water sensitive cash crops, reduce water consumption by 30-70%, and lower fertilizer usage by up to 40% [1–5]. Advancements in irrigation systems are crucial to alleviating farmer poverty while increasing worldwide food and water security. Globally, 70% of the 2.5 billion people who depend on smallholder farming for sustenance live in abject poverty, surviving on only $2 a day [6, 7]. Drip irrigation is a promising means of increasing agricultural yields, and numerous cross-country studies have found a positive correlation between increased agricultural productivity and poverty reduction [6, 8, 9]. Other studies have shown that agricultural growth is fives times more effective at poverty reduction than growth in other sectors [10]. Currently, the price of drip irrigation, particularly in off-grid settings which require solar or diesel power (where the majority of poor farmers live) prevent the technology from large-scale adoption [5, 11]. The aim of this paper is to parametrically describe emitter design and performance, and provide engineers with design tools which can be used to create improved drip systems. These tools may be used to optimize new emitter architectures with lower pressure, power, and cost, which could open up new markets in poor, smallholder farming communities.

**Fig 1:**
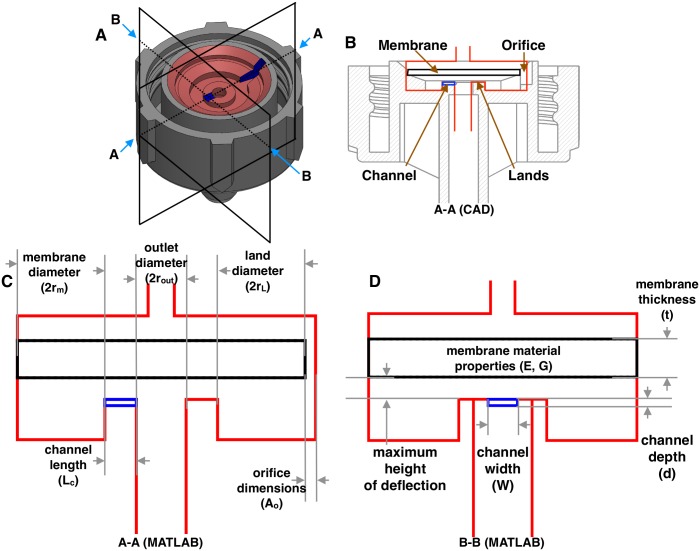
Schematics of a conventional pressure compensating online drip emitter which uses a flexible membrane to control flow rate. A: Isometric view showing the section planes. B: Cross-sectional view on the A-A plane. C: MATLAB modeled schematic corresponding to the cut view on the A-A plane. D: MATLAB modeled schematic corresponding to the cut view on the B-B plane. C and D show the critical dimensions of the flow features within the emitter which were used to model its behavior.

PC drip emitters were popularized in Israel in the 1960s [12]. The first patent for a flow regulating device similar to the common drip emitter architecture investigated in this study (Fig 1) was patented in 1952 [13]. To the authors’ knowledge, there is no existing study that analytically describes the principal operating characteristics and their dependence on geometric features for PC drip emitters. Fig 1 shows the important parameters that influence the performance of these devices. The analysis presented in this study may enable engineers to optimize new emitter architectures with a lower activation pressure, which would require less power to pump water through the irrigation system while maintaining uniform distribution.

**Fig 2:**
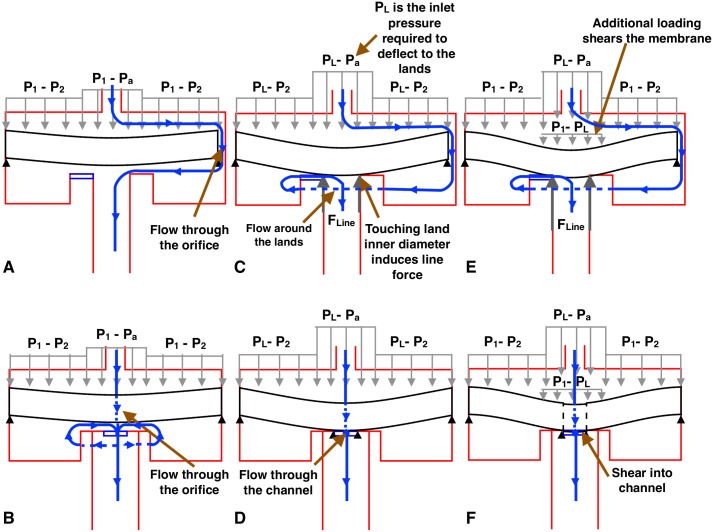
Graphical summary of the working principle of a drip emitter. A and B: Bending of the flexible membrane shown in the A-A and B-B planes from Fig 1A, respectively. C and D: Line force contact between the membrane and the lands, shown in the A-A and B-B planes from Fig 1A, respectively. E and F: Deflection of the membrane into the channel from shearing, shown in the A-A and B-B planes from Fig 1A, respectively. The flow path of water is shown by the blue lines and arrows. The dashed blue lines signify fluid flow behind the objects in view. Gray arrows denote the pressure differential acting on the membrane. Bold arrows denote the contact force at the edge of the land, *F*_*Line*_. The black triangles show constraints to membrane deflection.

The type of emitter shown in Fig 1 is called an ‘online’ emitter, which is installed on the outside of irrigation tubing; in contrast, ‘inline’ emitters are molded directly into the inside of the tubing. The qualitative working principle of an online emitter (Fig 2) are as follows. Fluid flows into the emitter through the inlet at pressure *P*_1_. The fluid then flows through an orifice into the chamber under the membrane. The flow through the orifice leads to a pressure loss, with the pressure in the chamber at *P*_2_. The fluid then flows out of the emitter to the atmosphere at pressure *P*_a_. The flow of fluid creates a pressure differential across the membrane. As the inlet pressure, *P*_1_, increases, the compliant membrane deflects down until it touches the lands (Fig 2C and 2D). The inlet pressure required to deflect the membrane to the lands is labelled *P*_L_. Once the inlet pressure increases to *P*_1_ ≥ *P*_L_, the fluid now has to flow around the chamber and through the channel to the outlet. As the inlet pressure increases further, the additional loading (*P*_1_—*P*_L_) results in the membrane shearing into the channel (Fig 2F). This membrane shearing increases with rising inlet pressure, resulting in a reduced area for the fluid flow path in the channel, and hence more flow resistance.

**Fig 3:**
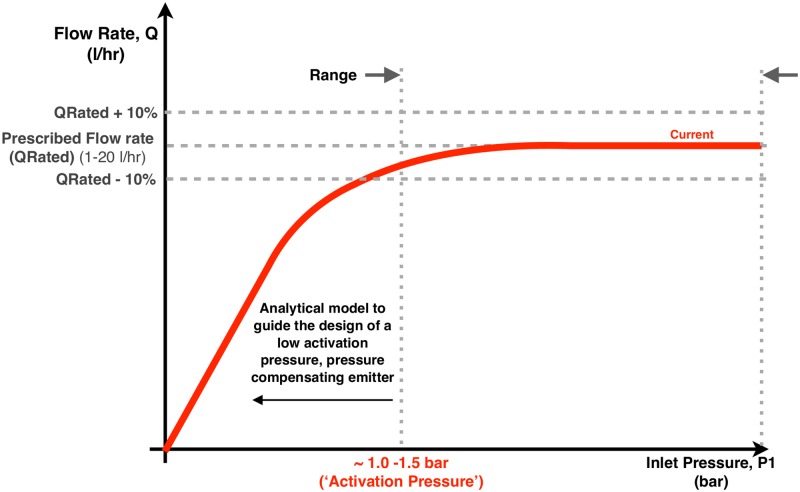
Flow control performance of a PC drip emitter. Solid line shows the generic behavior of a commercially available PC emitter [14], which was used as the benchmark in this study. The leftmost vertical dotted line shows the ‘activation pressure’, which is the minimum pressure required for the emitter to achieve its rated volumetric flow rate. The region marked ‘Range’ denotes the typical operation pressure range for the emitter. The horizontal dashed lines show acceptable tolerances for the rated volumetric flow rate.

PC emitters deliver a constant flow rate over a specified operating pressure range. Their performance is characterized by the rated volumetric flow rate versus applied inlet pressure. Fig 3 shows the performance of a currently manufactured drip emitter [14], which was the benchmark used in this study. The leftmost vertical dotted line shows the activation pressure.

This paper begins with describing a high fidelity but simple coupled fluid-solid mechanics model for emitter behavior. It then describes the methodology and prototypes used to validate the fidelity of this model. This is followed by a discussion section that identifies the key insights gained from the analytical model and the validation.

## Parametric modeling

### Coupled fluid-solid mechanics solver

**Fig 4:**
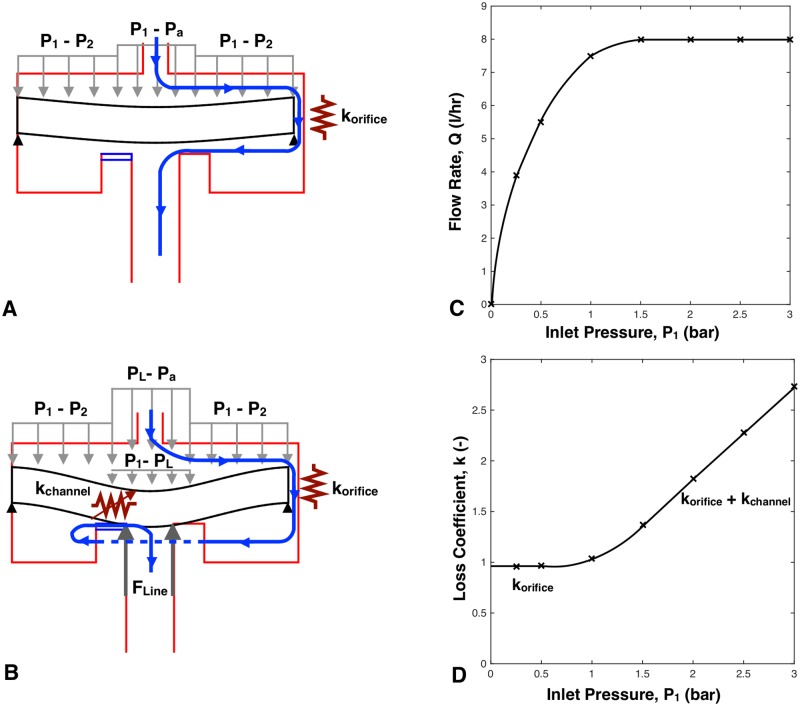
Fluid flow modeling through an 8 L/hr drip emitter. A: Bending of the flexible membrane under initial loading, cut in the A-A plane shown in Fig 1A. The primary flow restriction in this case is caused by *κ*_*orifice*_, shown by a resistor symbol and plotted in the first section of Fig 4D. B: Shearing of the flexible membrane into the channel, cut in the A-A plane shown in Fig 1. Flow restriction is caused by the sum of *κ*_*orifice*_ and the variable resistance (shown by the variable resistor symbol) of *κ*_*channel*_, which increases with rising inlet pressure as shown in Fig 4D. C: Flow rate versus inlet pressure for pressure compensating behavior. D: Loss coefficient in the fluid network versus inlet pressure.

**Fig 5:**
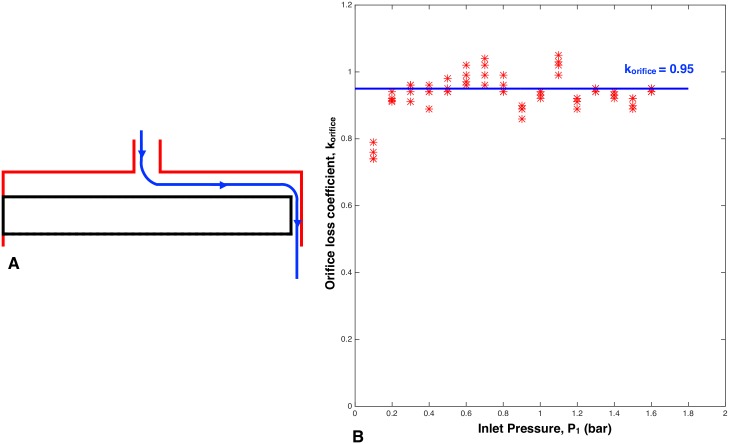
Measurement of *κ*_*orifice*_. A: Cross-section schematic of a modified emitter used to measure the orifice loss coefficient. The bottom half of a conventional emitter (like that in Fig 1) was removed so the orifice was the only source of flow restriction. For this test, the compliant membrane was replaced by a solid membrane. B: Measured values of *κ*_*orifice*_ versus pressure, with an average of *κ*_*orifice*_ = 0.95.

Fig 4 shows the schematic of the fluid circuit within a PC emitter which was used in this study. The circuit was modeled as two flow resistors in series (Fig 4B). One resistance occurs from the fluid flow through the orifice, *κ*_*orifice*_, and was experimentally found to be 0.95 (Fig 5). The other resistor is variable due to channel flow modeled using the Darcy-Weisbach equation [15–18], where changes in pressure affect the deformation of the membrane and hence the length and cross-sectional area of the channel flow path.

Bending of the circular silicone membrane was modeled using Kirchhoff Love plate theory and then superimposing a large deflection correction factor. This factor was empirically derived by Timoshenko for metal circular plates and then expanded to other materials using Poisson ratio correlations [19]. The plate shearing into the channel was modeled by the shearing of a wide beam, as the ratio of length to width of the section of silicone membrane pushed into the channel is always greater than 5:1 for the pressure range of the emitter.

Due to the steady nature of the silicone membrane deformation and the fluid flow through the emitter, the coupled fluid-solid mechanics was characterized using an iterative modeling technique [20–23]. At every inlet pressure, the structural deformation was solved analytically from a set of steady-state equations as described in the subsection Structure Deformation of the Membrane. The membrane deformation and the emitter body define the fluid flow paths. The fluid flow was concurrently solved analytically from a set of steady state equations described in the subsection Fluid Flow Modeling. The resulting pressure differential applied to the membrane due to flow resistance was then compared to the assumed pressure differential applied in the structural deformation model. This coupled problem was solved iteratively until the pressure differential predicted from both sets of equations reached an agreement within 1%. This iterative process is further explained in The Iterative Solver subsection.

### The iterative solver

**Fig 6:**
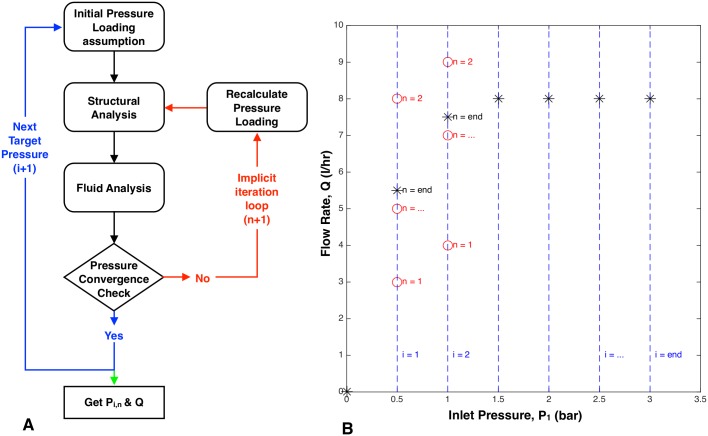
Iterative process used to model the coupled fluid-structure behavior within a drip emitter. A. Block diagram of the solver. B. The flow rate is solved iteratively for each increment of inlet pressure. The vertical dashed lines correspond to input pressure steps in the model. The circles are iterative solutions to flow rate. The asterisks are the final solution of flow rate for each inlet pressure. This model results in the full pressure versus flow rate relationship for a emitter of given internal geometry and membrane material.

Fig 6 graphically depicts the iterative process used to solve the coupled fluid-structure system in this study. The output of the iteration is an inlet pressure versus flow rate curve that depends on the fluid flow path geometry and membrane materials within the emitter. For every increment of the inlet pressure (vertical dashed lines in Fig 6), the model iteratively calculates the flow rate out of the emitter (asterisks in Fig 6).

The iterative solver uses the following process. For every inlet pressure *P*_1_ applied to the emitter, an initial pressure loading of *P*_2_ = *P*_a_ is assumed. The membrane deflection is then calculated using this pressure loading. The resulting membrane deflection defines the flow path through the emitter and is used to determine the resulting flow rate, *Q*_n = 1_. Finally, *Q*_n = 1_ is used to recalculate *P*_2_ with

$$\begin{array}{c} P_{2}^{n+1}=P_{1}^{i}-\frac{1}{2}\rho {\left(\frac{Q^{n}}{A_{orifice}}\right)}^{2}\kappa_{orifice}. \end{array}$$ (1)

Here, *κ*_*orifice*_ = 0.95 is the experimentally measured loss coefficient for the orifice in an 8 L/hr commercial emitter (Fig 5B), and *A*_*orifice*_ is the orifice area. The recalculated *P*_2_^n+1^ is used to update the deflection and the flow rate, *Q*^n+1^. This iterative process is repeated until the flow rate from the previous iteration and the new flow rate converge to within 1% ($\frac{Q^{n+1}-Q^{n}}{Qn}\leq 0.01$). A convergence threshold of 1% was set based on the observed computational time for the overall algorithm. This iterative process is repeated for each value of inlet pressure, *P*_1_^i+1^, to build the entire flow rate versus pressure curve (Fig 6).

### Structure deformation of the membrane

The deformation of the circular membrane can be characterized by three regimes. As the inlet pressure is increased, first the compliant membrane deforms in bending up to the lands (Fig 2A and 2B). The membrane then contacts the lands and seals the channel (Fig 2C and 2D). Finally, it deforms in shear into the channel (Fig 2E and 2F).

#### Deformation in bending

The small deflection in bending, *w*, was given by the solution of the fourth order partial differential equation [24]

$$\begin{array}{c} \nabla^{4}w=\frac{q}{D}, \end{array}$$ (2)

where

$$\begin{array}{c} D=\frac{Et^{3}}{12(1-\nu^{2})} \end{array}$$ (3)

and *q* is the pressure loading, *D* is the flexural stiffness of the membrane, *E* is the Young’s modulus, *t* is the thickness, and ν is the poisson ratio of the membrane. The assumed pressure loading is shown in Fig 2A and 2B. The circular membrane was assumed to be thin and simply-supported along the outer edge. These boundary conditions reflect that the membrane can physically move radially while supporting rotation along the edge.

Because the geometry of, and loading on, the membrane are axisymmetric, the spatial coordinate system can be simplified to 2D, perpendicular and transverse to the circular plate. The deflection due to loading in Fig 2A and 2B can be calculated by assuming the deflections as small and linear. They are determined by superimposing the deflection of a circular plate due to uniform loading from *P*_1_ − *P*_*a*_, and then subtracting the deflection due to annular loading from *P*_2_ − *P*_*a*_. The exact analytical solutions for these loadings are known and given by [24]. Deflection of a circular plate due to uniform loading is

$$\begin{array}{c} w_{uniform}\left(r\right)=\left(P_{1}-P_{a}\right)\frac{r_{m}^{4}}{64D}\left(1-{\left(\frac{r}{r_{m}}\right)}^{2}\right)\left(\frac{5+\nu }{1+\nu }-{\left(\frac{r}{r_{m}}\right)}^{2}\right), \end{array}$$ (4)

where *r*_m_ is the membrane radius and *r* is a spatial position in the radial direction. Deflection of circular plate due to annular loading is

$$\begin{array}{c} w_{annular}\left(r\right)=-\left(P_{2}-P_{a}\right)\frac{r_{m}^{4}}{2D}\left(\frac{L_{17}}{1+\nu }-2L_{11}\right)+\frac{\left(P_{2}-P_{a}\right)r_{m}^{2}L_{17}r^{2}}{2D(1+\nu )}-\frac{\left(P_{2}-P_{a}\right)r^{4}G_{11}}{D}, \end{array}$$ (5)

where for r < r_p_

$$\begin{array}{c} G_{11}=0, \end{array}$$ (6)

and for r > r_p_

$$\begin{array}{c} G_{11}=\frac{1+4{\left(\frac{r_{p}}{r}\right)}^{2}-5{\left(\frac{r_{p}}{r}\right)}^{4}-4{\left(\frac{r_{p}}{r}\right)}^{2}\left(2+{\left(\frac{r_{p}}{r}\right)}^{2}\right)\log\left(\frac{r}{r_{p}}\right)}{64}. \end{array}$$ (7)

For all r

$$\begin{array}{c} Y_{c}=-\frac{\left(P_{2}-P_{a}\right)r_{m}^{4}}{2D}\left(\frac{L_{17}}{1+\nu }-2L_{11}\right), \end{array}$$ (8)

$$\begin{array}{c} M_{c}=\left(P_{2}-P_{a}\right)r_{m}^{2}L_{17}, \end{array}$$ (9)

$$\begin{array}{c} L_{17}=\frac{1-\frac{1-\nu }{4}\left(1-{\left(\frac{r_{p}}{r_{m}}\right)}^{4}\right)-{\left(\frac{r_{p}}{r_{m}}\right)}^{2}\left(1+\left(1+\nu \right)\log\left(\frac{r_{m}}{r_{p}}\right)\right)}{4},\text{and} \end{array}$$ (10)

$$\begin{array}{c} L_{11}=\frac{1+4{\left(\frac{r_{p}}{r_{m}}\right)}^{2}-5{\left(\frac{r_{p}}{r_{m}}\right)}^{4}-4{\left(\frac{r_{p}}{r_{m}}\right)}^{2}\left(2+{\left(\frac{r_{p}}{r_{m}}\right)}^{2}\right)\log\left(\frac{r_{m}}{r_{p}}\right)}{64}, \end{array}$$ (11)

where *r*_p_ is the radial position of the start of the distributed annular loading and is equivalent to half way between the lands ((*r*_out_ + *r*_L_)/2). *G*_11_ is a function of the radial location *r* and the start of annular loading *r*_p_. *Y*_c_ is the center deflection and *M*_c_ is the center moment. *L*_11_ and *L*_17_ are loading constants dependent on the ratio of *r*_p_/*r*_m_. The total deflection is the summation of the two loadings,

$$\begin{array}{c} w_{bending}\left(r\right)=w_{uniform}\left(r\right)+w_{annular}\left(r\right). \end{array}$$ (12)

Eqs (4) through (12) are valid for small deflections (in this case if the maximum bending deflexion *w*_*bending*,*max*_ < *t*). Once the deflections exceed the thickness of the plate, induced radial stresses cause the plate to stiffen, and the deflection is no longer proportional to the magnitude of the loading.

Timoshenko derived a correction that accounts for the stiffening effect, which can be multiplied by the small deflection to give a good estimate of the actual deflection [19]. The correction factor is

$$\begin{array}{c} \frac{w_{max,large}}{t}+0.262{\left(\frac{w_{max,large}}{t}\right)}^{3}-\frac{w_{max,small}}{t}=0, \end{array}$$ (13)

$$\begin{array}{c} w_{bending,large}\left(r\right)=w_{bending,small}\left(r\right)\left(\frac{w_{max,large}}{w_{max,small}}\right), \end{array}$$ (14)

where *w*_max,large_ is the maximum large deflection, and is dependent on the thickness of the membrane and the maximum small deflection for a loading, *w*_max,small_.

Once the largest deflection under small-scale deformations (*w*_*max*,*small*_) is determined, Eq (13) is solved to find the largest deflection under large deflection assumptions (*w*_*max*,*large*_). The ratio $\frac{w_{max,large}}{w_{max,small}}$ is then used to scale the calculated deformation under the small deformation assumption to large deflection where *w*_*bending*,*max*_ > *t* (Eq (14)).

#### Deformation while interacting with the lands

Once the membrane deflects to the lands, an opposing circular line force is induced to prevent the membrane from deflecting further (Fig 2C and 2D). This line force is due to the contact of the membrane on the inner diameter of the lands. The magnitude of the force is derived by employing the boundary condition that the membrane cannot deflect further than the lands. The pressure required to cause the membrane to deflect to the lands is labelled *P*_L_.

#### Deformation in shear

Once the membrane deflects to the lands, additional pressure loading of *P*_1_ − *P*_*L*_ will result in membrane deflection in shear into the channel (Fig 2E and 2F).

The section of membrane that deflects into the channel is small, with a thickness to length ratio of ∼1. The deformation is approximated using thick beam hyperbolic shear deformation theory [25]. The governing equations are

$$\begin{array}{c} EI\frac{d^{4}w}{dx^{4}}-EIA_{0}\frac{d^{3}\phi }{dx^{3}}=q,\;\text{and} \end{array}$$ (15)

$$\begin{array}{c} EIA_{0}\frac{d^{3}w}{dx^{3}}-EIB_{0}\frac{d^{2}\phi }{dx^{2}}+GAC_{o}\phi =0. \end{array}$$ (16)

The boundary conditions are: no deflection into the channel at the channel edges; the rotation of the membrane at the edges of the channel must match the rotation of the rest of the membrane at the same radial position. The portion of the membrane spanning the channel was approximated to have clamped-clamped beam end conditions [25], yielding shear deflection of

$$\begin{array}{c} w_{shear}\left(x\right)=\frac{3}{5}\frac{\left(P_{1}-P_{L}\right)W^{2}}{GA_{b}}\left(\frac{x}{W}-\frac{x^{2}}{W^{2}}-\left(\frac{\cosh\left(\lambda \frac{W}{2}\right)-\cosh\lambda \left(\frac{W}{2}-x\right)}{\lambda L\sinh(\lambda \frac{W}{2})}\right)\right), \end{array}$$ (17)

where

$$\begin{array}{c} \lambda^{2}=\frac{\beta }{\alpha };\alpha =\frac{B_{0}}{A_{0}}-A_{0};\beta =\frac{GA_{b}C_{0}}{EIA_{0}}, \end{array}$$ (18)

$$\begin{array}{c} A_{0}=\cosh\left(\frac{1}{2}\right)-12\left(\cosh\left(\frac{1}{2}\right)-2\sinh\left(\frac{1}{2}\right)\right), \end{array}$$ (19)

$$\begin{array}{c} B_{0}=\cosh^{2}\left(\frac{1}{2}\right)+6\left(\sinh\left(1\right)+1\right)-24\cosh\left(\frac{1}{2}\right)\left(\cosh\left(\frac{1}{2}\right)-2\sinh\left(\frac{1}{2}\right)\right),\;\text{and} \end{array}$$ (20)

$$\begin{array}{c} C_{0}=\cosh^{2}\left(\frac{1}{2}\right)+\frac{1}{2}\left(\sinh\left(1\right)+1\right)-4\cosh\left(\frac{1}{2}\right)\sinh\left(\frac{1}{2}\right). \end{array}$$ (21)

In Eqs (17)–(21), *G* is the shear modulus of the membrane, *A*_b_ is the cross-sectional area of the beam, *W* is the length of beam and width of channel, and *x* is the spatial position which is perpendicular to the radial position r (*x* represents the distance along the width of the channel whereas *r*—*r*_*out*_ is representative of the distance along the channel length). Constants *A*_0_, *B*_0_ and *C*_0_ appear in the coupled Euler-Largrange governing differential equations of a thick beam deforming in bending and shear [25].

#### Total deformation

The calculated membrane deformations *w*_*bending*_(*r*) and *w*_*shear*_(*x*) are relative to different coordinate systems. To find the total deflection of the membrane into the channel, the deflection due to bending *w*_*bending*_(*r*) is added to the deflection due to shear *w*_*shear*_(*x*) within the local *r*, *x*, *z* coordinate system within the channel, where *w* acts in the *z* direction.

### Fluid flow modeling

The fluid flow was modeled using a set of steady-state equations that capture the dominant pressure losses in the emitter caused by flow through the orifice (Fig 4A) and through the channel (Fig 4B).

#### Pressure loss through the orifice

The pressure drop due to the orifice is calculated by

$$\begin{array}{c} \Delta P_{orifice}=P_{1}-P_{2}=Q^{2}\left(\frac{1}{2}\frac{\rho }{A_{orifice}^{2}}\kappa_{orifice}\right), \end{array}$$ (22)

where *κ*_*orifice*_ = 0.95 is the experimentally obtained loss coefficient value for the orifice in the 8 L/hr benchmark dripper used in this study (Fig 5B).

#### Pressure loss through the channel

Studies [15–18] have shown that the pressure drop within a channel with length scales of the order of hundreds of microns can be evaluated using macro-scale formulae. Hence the pressure drop and flow rates through the channel can be expressed by

$$\begin{array}{c} \Delta P_{channel}=P_{2}-P_{a}=\frac{1}{2}\rho \frac{Q^{2}}{A_{channel}^{2}}\left(\kappa_{inlet}+\kappa_{friction}+\kappa_{outlet}\right). \end{array}$$ (23)

This is rearranged to give

$$\begin{array}{c} \Delta P_{channel}=Q^{2}\left(\frac{1}{2}\frac{\rho }{A_{channel}^{2}}\left(\kappa_{inlet}+\frac{fL}{D_{h}}+\kappa_{outlet}\right)\right), \end{array}$$ (24)

where

$$\begin{array}{c} D_{h}=\frac{4A_{channel}}{p_{channel}} \end{array}$$ (25)

is the equivalent hydraulic diameter, *A*_channel_ is the area of the channel, *p*_channel_ is the perimeter of the channel, *f* is the friction factor, *ρ* is the fluid density, *Q* is the volumetric flow rate through the emitter, *L* is the effective length of channel (the portion of the channel covered by the membrane (i.e. the portion of channel through which the fluid must flow), and *κ*_inlet_ and *κ*_outlet_ are the minor loss coefficients due to inlet and exit from the channel, respectively, whose values can be obtained from Kays and London [26].

The friction factor for laminar flow (i.e. for *Re*_D_h__ < 2000) can be calculated as

$$\begin{array}{c} f=\frac{N}{Re_{D_{h}}}, \end{array}$$ (26)

where *N* is dependent on the cross-sectional aspect ratio of the channel [27]. For *Re*_D_h__ > 3000 (i.e. assumed turbulent flow), the friction factor can be calculated using the Colebrook equation despite the non-circular cross section [28],

$$\begin{array}{c} \frac{1}{\sqrt{f}}=-2\log_{10}\left(\frac{\epsilon }{3.7D_{h}}+\frac{2.51}{Re_{D_{h}}\sqrt{f}}\right), \end{array}$$ (27)

where *ϵ* is the roughness of the flow path and the flow is usually in the turbulent regime.

The flow rate can be calculated using Eqs (23)–(27) and mass continuity for incompressible fluids. Using this calculated flow rate, the pressure loading can be recalculated and the iteration described in Fig 6 can be repeated.

#### Total pressure loss through the emitter

To solve Eqs (22)–(27), the area of orifice (*A*_*orifice*_), orifice loss coefficient (*κ*_*orifice*_), the channel area (*A*_*channel*_), the channel perimeter (*p*_*channel*_), and the effective length of channel (*L*) must be determined. *A*_*orifice*_ remains constant with changes in pressure, as the orifice geometry is unaffected by the membrane deformation. *κ*_*orifice*_ was experimentally found to be constant (Fig 5B). *A*_*channel*_, *p*_*channel*_, and *L* are all functions of the membrane deformation. From the Structure Deformation of the Membrane subsection, membrane deflection *w*(*r*, *x*) can be determined. *L* is the length of the membrane that fully covers the lands, which can be calculated as the radial distance between the innermost position on the membrane where *w*_*shear*_ = 0 (provided *P*_1_ > *P*_*L*_) and the inner radius of the lands.

In the Structure Deformation of the Membrane subsection, it was noted that the deformation of the circular membrane is split into three regimes. These three regimes result in two distinct flow conditions within the emitter (Fig 2). When the inlet pressure is low and in the regime of bending the membrane down to the lands, the major pressure losses occur within the orifice, given by

$$\begin{array}{c} \Delta P_{total}=P_{1}-P_{a}=\Delta P_{orifice}=Q^{2}\left(\frac{1}{2}\frac{\rho }{A_{orifice}^{2}}\kappa_{orifice}\right). \end{array}$$ (28)

As the membrane approaches the lands the effective loss coefficient of the orifice will rise, even before the membrane touches the lands (seen in transition region in Fig 4D). It was experimentally observed that this rise in orifice loss coefficient is less than 3%, and was thus neglected in the following calculations.

When the inlet pressure increases and the membrane touches the lands and starts to shear into the channel, the second flow regime begins. Pressure drop in the emitter is now caused by the orifice and the channel, described by

$$\begin{array}{ccc} \Delta P_{total} & = & P_{1}-P_{a}=\Delta P_{orifice}+\Delta P_{channel}=Q^{2}\left(\frac{1}{2}\frac{\rho }{A^{2}}\kappa_{total}\right)\\ & = & Q^{2}(\frac{1}{2}\frac{\rho }{A_{orifice}^{2}}\kappa_{orifice}+\frac{1}{2}\frac{\rho }{A_{channel}^{2}}\left(\kappa_{inlet}+\frac{fL}{D_{h}}+\kappa_{outlet}\right). \end{array}$$ (29)

### Explanation of PC behavior

Eqs (28) and (29) summarize how the the flow rate is dependent on the inlet pressure. Fig 4D provides insight into the fact that to achieve pressure-compensating behavior, the total loss coefficient, *k*_total_, needs to vary linearly with the increase in inlet pressure. Fig 4D shows two trends. Before the pressure compensating regime, *k*_total_ is approximately constant at 0.95, which is the experimentally measured value of *k*_orifice_. This confirms that before the membrane touches the lands, the major pressure losses occur due to flow restriction through the orifice. Pressure compensation begins when the flexible membrane shears into the channel, creating an additional increase in the overall resistance by adding the variable resistance *k*_channel_.

Eq (29) can be rewritten as

$$\begin{array}{c} \Delta P_{total}=Q^{2}\left(K_{orifice}+K_{minor}+K_{friction}\right), \end{array}$$ (30)

where

$$\begin{array}{c} K_{orifice}=\frac{1}{2}\frac{\rho }{A_{orifice}^{2}}\kappa_{orifice}, \end{array}$$ (31)

$$\begin{array}{c} K_{minor}=\frac{1}{2}\frac{\rho }{A_{channel}^{2}}\left(\kappa_{inlet}+\kappa_{outlet}\right),\text{and} \end{array}$$ (32)

$$\begin{array}{c} K_{friction}=\frac{1}{2}\frac{\rho }{A_{channel}^{2}}\frac{fL}{D_{h}}. \end{array}$$ (33)

**Fig 7:**
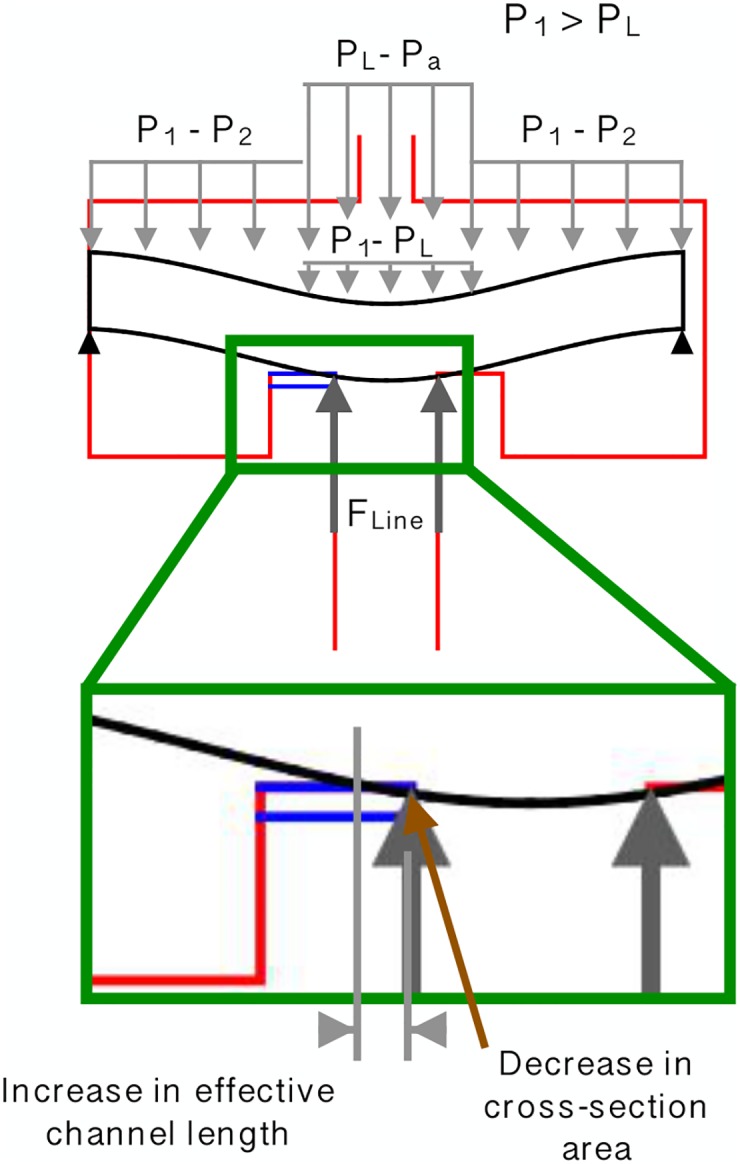
Mechanics that yield pressure compensating behavior and a linear increase in the total loss coefficient. Bending of the flexible membrane under loading, cut in the A-A plane from Fig 1A. Increases in inlet pressure cause the flexible membrane to deflect further and cover up a larger length of the channel. This results in an increase in effective length of the flow path. As the membrane shears further into the channel with increasing input pressure, the cross sectional area and hydraulic diameter of the channel are decreased.

*K*_minor_ is dependent on the inlet and outlet loss coefficients, which are in turn dependent on the Reynolds number. These have been assumed to be constant in the turbulent regime. Assuming the friction factor *f* does not change significantly in the turbulent regime encountered (see Table 1 for calculated values of *f*), Eq (33) shows that for the emitter to exhibit pressure compensating behavior, *K*_friction_ needs to increase linearly with pressure. For *K*_friction_ to increase, the effective length of the channel must increase (Fig 7A), and the hydraulic diameter and cross-sectional area of the channel must decrease (Fig 7B). These effects vary in combination to achieve a linearly increasing *K*_friction_, and thus pressure compensation with increasing input pressure.

**Table 1:** Calculated geometric changes in the channel and resulting friction factor with increasing pressure.

Pressure	*L*(*mm*)	*D*_*h*_(*mm*)	*A*_*channel*_(*mm*^2^)	*Re*_*channel*_	*f*
1 bar	0.28	0.295	0.173	3258	0.044
2 bar	0.36	0.253	0.145	3997	0.041
3 bar	0.52	0.210	0.117	4094	0.041

Note that *D*_*h*_, *Re*_*channel*_, and *A*_*channel*_ are calculated based on the values at the output of the channel. These may vary along the channel, which can be accounted for by discretizing the effective length of channel, *L*. The effective length is considerably smaller than the channel feature in the 8 L/hr dripper used in this study, which is 2.40 mm long.

## Experimental methods

**Fig 8:**
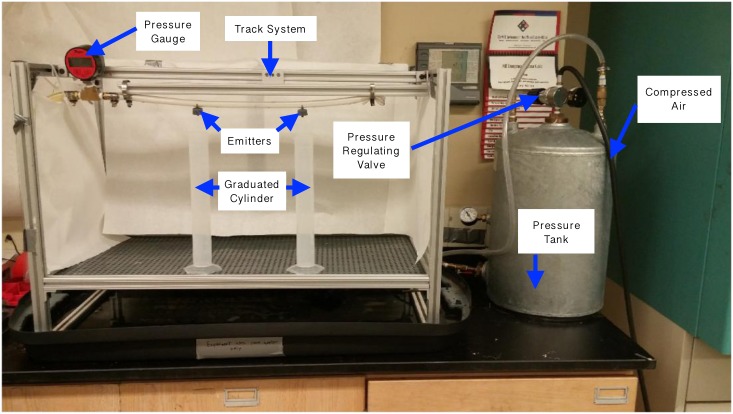
Experimental setup used to test drip emitters. The inlet pressure of water connected to the emitters is controlled by a pressure regulating valve. Flow rate was determined by measuring the time to fill 250 ml graduated cylinders. Two drip emitters could be tested simultaneously.

The experimental setup used to validate the theoretical models developed in the Parametric Modeling section is shown in Fig 8. The setup was designed to measure pressure versus flow rate behavior of drip emitters, to form graphs like that in Fig 3 which can be compared to manufacturers’ published data. The setup is a scaled-down version of the apparatus used to characterize drip emitter behavior in industry, as well as the setup described in the Irrigation Training and Research Center technical report of 2013 [29]. This similarity ensured that the data collected was comparable to data available for commercial emitters. The emitter testing setup was comprised of three main components: a pressurized tank, the test bench, and a flow measurement system.

The water supply was held in the pressurized tank, which was regulated to 2 bar static head using compressed air. This water supply was run through a pressure regulating valve to lower the inlet pressure to the required testing target pressure. A pressure gauge was used to measure the actual pressure applied to the emitters.

The experimental setup mimicked the piping and emitter setup in a field and enabled two emitters to be tested simultaneously. The water from the water supply was feed into the test bench through a 0.5 inch pipe. This pipe is mounted on a movable track. The movable track ensures that when the timer starts, both emitters can be moved into place over the graduated cylinders together; when the timer stops, they can be moved back simultaneously. This ensures flow rate readings are representative and repeatable. The emitters were mounted 12 in apart, as recommended by [29]. The system was operated by first bringing the tank up to pressure and starting flow through the emitters. After maintaining the target pressure for one minute, the track was shifted so the emitters dripped water into 250 mL graduated cylinders. Flow rate was derived by measuring the time required to fill the graduated cylinders.

The experimental protocol used in this study to test and characterize drip emitters followed the Indian Standard for Irrigation Equipment and Emitter Specification [30]. The resulting data enables flow rate versus pressure curves to be constructed, like that in Fig 3. Each dripper test included the following:

1. Pressurize the sample of emitters to be tested under one bar for five minutes
2. Adjust the hose pressure to 0.2 bar. Let the water from the emitters collect in a drainage tank underneath.
3. Allow the flow rate to stabilize (this will take one minute)
4. Once the pressure and flow rates have stabilized, move the emitters along the movable track to discharge into individual graduated cylinders. Simultaneously start a timer.
5. Time how long it takes to fill up the 250 ml graduated cylinders. Once full, move the emitters back to the starting position such that they are discharging into the drainage tank
6. Empty the graduated cylinders
7. Increase the hose pressure by 0.1 bar.
8. Repeat steps (3) through (7) until the pressure is at 1.6 bar, while recording both the pressure and the flow rates for the individual emitters.
9. Repeat the measurements for decreasing pressure levels starting at 1.6 bar and ending at 0.2 bar with 0.1 bar intervals.
10. Repeat increasing and decreasing pressure tests again.

The maximum pressure tested in this study was 1.6 bar; industrial tests go up to 3.0 bar. The tests in this study had to be performed at a lower pressure because of limitations in the experimental setup. The pressurized tank used was rated to a maximum of 2.0 bar, and a 20% safety factor was applied. Furthermore, the machined prototype emitters tested in this study leaked at pressures higher than 1.3 bar because they did not include the sealing surfaces found in commercially-available, injection molded emitters.

### Measurement uncertainty

The pressure applied to the emitters was adjusted based on visual reading of a digital pressure gauge in Fig 8. The resolution of the gauge is ± 0.02 bar. The graduated cylinders used to measure the volume had a resolution of 2 mL and a measurement capacity of 250 mL, resulting in an uncertainty of 250 ± 2 mL. The timer relied on human reaction time, which had an uncertainty of ± 0.25 s. Given these factors, the flow rate readings in the range that was measured had an error due to measurement uncertainty of less than 2%, and the pressure readings had an error of less than 10%. For an example of how error varied during tests, Table 2 presents a detailed error analysis on the experimental raw data obtained from the commercially manufactured 8 L/hr emitter.

**Table 2:** Error analysis on experimental measurements.

Pressure (bar)	0.2	0.4	0.6	0.8	1	1.2	1.4	1.6
Resolution (bar)	0.02	0.02	0.02	0.02	0.02	0.02	0.02	0.02
Relative error (%)	10.0	5.0	3.3	2.5	2.0	1.7	1.4	1.3
Volume (ml)	250	250	250	250	250	250	250	250
Resolution (ml)	2	2	2	2	2	2	2	2
Relative error (%)	0.8	0.8	0.8	0.8	0.8	0.8	0.8	0.8
Time(s)	400	189	144	129	120	115	113	113
Resolution (s)	0.25	0.25	0.25	0.25	0.25	0.25	0.25	0.25
Relative error (%)	0.1	0.1	0.2	0.2	0.2	0.2	0.2	0.2
Flow rate (l/h)	2.3	4.8	6.3	7.0	7.5	7.8	8.0	8.0
Relative error (%)	0.81	0.81	0.82	0.82	0.82	0.82	0.82	0.82

The experimental raw data used for this analysis is from the commercially manufactured 8 L/hr emitter

### Parameteric study

Fig 1C and 1D show ten variables that influence a drip emitter’s performance. Out of these, the channel width, length, and depth, and the maximum height of deflection were chosen to perform a parametric study. The parameters of the silicone membrane were not varied, as it is common to use the same membrane in emitters of different rated flow rates. The silicone membrane is injection molded to shape and the material properties, used in this study, were measured as: Young’s modulus (*E*) = 0.038 GPa; shear modulus (*G*) = 0.60 MPa; and Poisson ratio (*ν*) = 0.48.

Nine different configurations of emitters (Table 3) were tested to validate the theory presented in the Parametric Modeling section. The benchmark used for this study was a commercially available 8 L/hr emitter made by Jain Irrigation Systems, Ltd. (labeled JAIN in Table 3). Emitter 1 was a fabricated prototype with geometry close to that of JAIN, and was used as a control. Due to the JAIN design being proprietarily, the dimensions of Emitter 1 are slightly different. Emitters 2 to 6 were fabricated such that only one parameter was changed at a time, in order to validate the predictive accuracy of the model for single variables. In Emitters 7 and 8, multiple variables were changed relative to the control in order to confirm the model is able to capture the interaction between variables.

**Table 3:** Dimensions for the nine emitters tested in this study.

	Emitters (mm)								
Parameter	JAIN	1	2	3	4	5	6	7	8
Channel Depth	ND	**0.30**	**0.35**	**0.45**	0.3	0.3	0.3	**0.25**	**0.15**
Channel Width	ND	**1.20**	1.20	1.20	**1.40**	1.20	1.20	1.20	**1.00**
Channel Length	ND	**2.40**	2.40	2.40	2.40	**4.80**	2.40	2.40	2.40
Max Height of Deflection	ND	**0.70**	0.70	0.70	0.70	0.70	**0.00**	**0.3**	0.70
Outlet Diameter	ND	**1.90**	1.90	1.90	1.90	1.90	1.90	1.90	1.90
Membrane Diameter	ND	**11.00**	11.00	11.00	11.00	11.00	11.00	11.00	11.00
Membrane Thickness	ND	**1.20**	1.20	1.20	1.20	1.20	1.20	1.20	1.20

The dimensions of JAIN emitter have not been disclosed (ND) because they are proprietary. Emitter 1 is the closest replica of the JAIN emitter, with slightly modified channel and land parameters. Emitter 1 was used as the control for this study. The bold and underlined values denote the changes made to the respective emitters when compared to the control.

Two identical emitters for each of the eight configurations shown in Table 3 were precision machined from delrin (polyoxymethylene) using a milling machine. The flow rate versus pressure curves for each of the emitters was found using the experimental setup and protocol described in this section. The experimental data were then compared with predictions from the theory presented in the Parametric Modeling section.

## Results and discussion

### Experimental data compared to theoretical predictions

**Fig 9:**
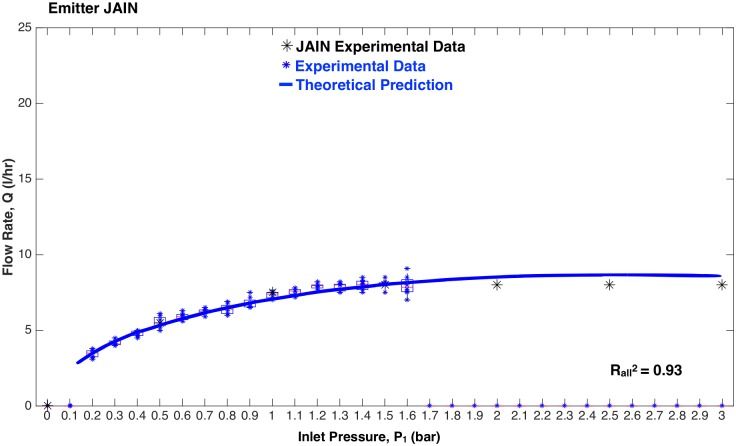
Flow rate versus inlet pressure for the JAIN emitter. Blue scatter dots are data collected in this study, aggregated as box plots. Black dots are results reported by the manufacturer. Solid line is theoretical prediction.

The flow rate versus inlet pressure results for the JAIN emitter and the eight prototypes are represented in Fig 9 through Fig 13. Each prototype has eight data points per pressure reading, as two identical emitters were tested while increasing and decreasing pressures during two trials. The experimental data are aggregated as box plots, with all of the raw data points shown as blue scatter dots. The theoretical prediction is plotted as a soild continuous line. The fit between the scatter dots and theory is reported as R^2^ values. Each graph shows two values of R^2^: ${\text{R}}_{1.3}^{2}$ was calculated based on experimental data up to 1.3 bar, and ${\text{R}}_{all}^{2}$ takes into account all the experimental data. This was done because at pressures greater than 1.3 bar, the machined emitters leaked as described earlier. The high pressure data points were included for completeness.

**Fig 10:**
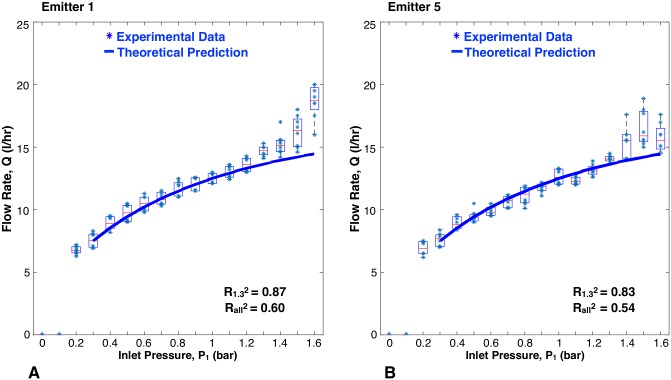
Flow rate versus inlet pressure for variations in channel length. Blue scatter dots are data collected in this study, aggregated as box plots. Solid line is theoretical prediction.

**Fig 11:**
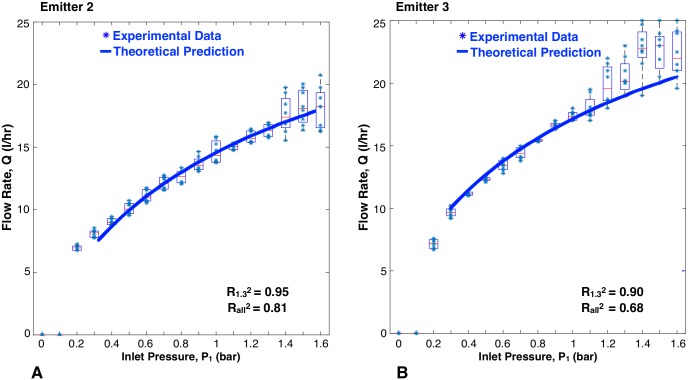
Flow rate versus inlet pressure with variations in channel depth. Blue scatter dots are data collected in this study, aggregated as box plots. Solid line is theoretical prediction.

**Fig 12:**
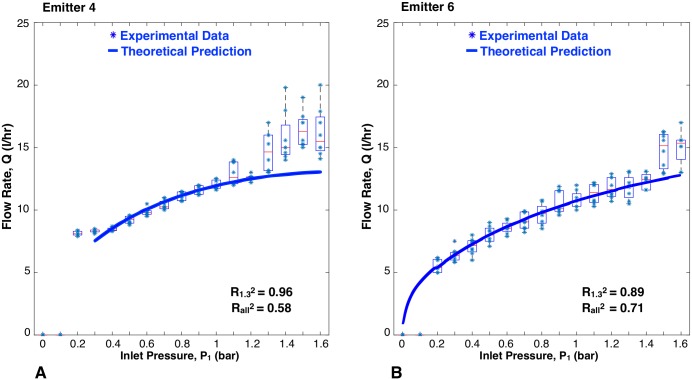
Flow rate versus inlet pressure with variations in topology. A: Variations in channel width; B: Variations in deflection to lands. Blue scatter dots are data collected in this study, aggregated as box plots. Solid line is theoretical prediction.

**Fig 13:**
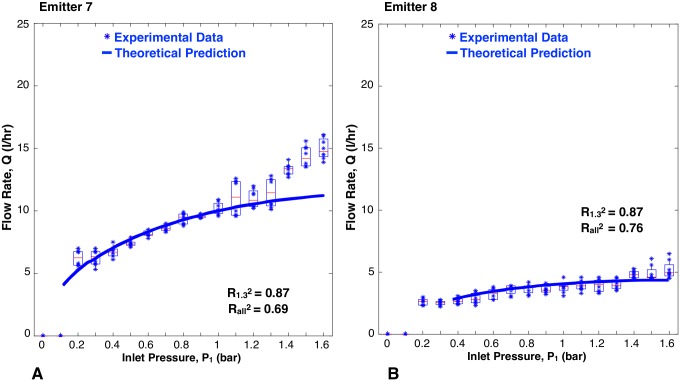
Flow rate versus inlet pressure for emitters with multiple geometric variations. A: Variations in both channel depth and max height of membrane deflection. B: Variations in both channel depth and channel width. Blue scatter dots are data collected in this study, aggregated as box plots. Solid line is theoretical prediction.

#### JAIN emitter

Fig 9 shows a close correlation between the experimental data and theoretical predictions for the JAIN emitter. The blue scatter dots are data collected by the authors during this study. The black dots are data published by the manufacturer. The ${\text{R}}_{all}^{2}$ value was calculated using both the blue and black scatter dots. Given the close fit between theory and experiment (with ${\text{R}}_{all}^{2}=0.93$), this plot shows the power of the parametric models presented in this study to predict pressure compensating behavior based on the internal geometry of the emitter.

#### Length of channel

Fig 10 shows a close correlation between the experimental data and theoretical predictions for differing channel lengths. For emitters 1 and 5, the ${\text{R}}_{1.3}^{2}$ values were 0.87 and 0.85, respectively. The trend seen is that an increase in channel length does not influence the flow rate versus pressure graph for the pressure range tested. This can be explained by the fact that the effective channel length (length of channel sealed by the membrane) does not vary at the pressures tested, hence there is no change in flow resistance. In both cases, the effective channel was significantly less than the length of the channel feature in the dripper (as explained in Table 1).

#### Depth of Channel

Fig 11 shows a close correlation between the experimental data and theoretical predictions for differing channel depths. For emitters 2 and 3, the ${\text{R}}_{1.3}^{2}$ values were 0.95 and 0.90, respectively. The trend seen is that an increase in channel depth led to increased flow rates. This trend is consistent with the discussion in the section. An increase in channel depth increases the cross-sectional area of the fluid flow path, leading to a decrease in flow resistance and higher flow rates. This trend is commonly seen in currently manufactured emitters where lower flow rate emitters have a shallower channel, while higher flow rate emitters have deeper channels [14, 31].

#### Width of channel

Fig 12A shows a good correlation between the experimental data and theoretical predictions for differing channel widths, with ${\text{R}}_{1.3}^{2}=0.96$. The trend seen is that an increase in channel width led to a decrease in flow rates, especially at higher inlet pressures. An increase in channel width facilitates larger deflection of the membrane in shear, which counteracts the increase in area.

#### Deflection to lands

Fig 12B shows a close correlation (${\text{R}}_{1.3}^{2}=0.89$) between the experimental data and theoretical predictions for emitter 6, which had a reduced max height of deflection between the membrane and the lands. The trend seen is that a decrease in deflection height of the membrane led to a decrease in flow rate. By reducing the height of deflection, the membrane will contact the lands and start shearing into the channel at a lower pressure, creating an increased flow resistance.

#### Multiple variables altered

Fig 13 shows a close correlation between the experimental data and theoretical predictions for emitter 7 (${\text{R}}_{1.3}^{2}=0.87$) and emitter 8 (${\text{R}}_{1.3}^{2}=0.87$), which both had multiple geometric variables altered. The trends of decreasing channel depth, increasing channel width, and decreasing the max height of membrane deflection all led to decreased flow rate, as predicted with the parametric models.

### Discussion

This paper provides four key insights into the design of PC drip emitters. First, to achieve pressure compensating behavior, the resistance to flow must increase linearly with pressure. Second, this linear increase in resistance can be induced by manipulating the area, length, and hydraulic diameter of the flow path. The resistance to flow is highly sensitive to the cross-sectional area of the flow path when compared to the length or hydraulic diameter. This relationship is currently exploited by manufacturers to create drippers of different flow resistance. Third, before the pressure compensating regime, the loss coefficient is fairly constant. This is because most of the pressure loss occurs through an orifice. To reduce activation pressure, the orifice loss coefficient should be reduced, which can be achieved by redesigning the shape of the orifice or increasing its area. Fourth, increasing channel depth, decreasing channel width, decreasing effective channel length, and increasing the maximum height of deflection of the membrane all lead to an increase in flow rate.

The experimental results validate the parametric model presented in this study, which can accurately predict the behavior of a PC drip emitter using a flexible membrane. The results show that the model is able to capture variations in all of the critical geometric factors within the emitter, and their impact on the flow rate versus pressure behavior. The insights gleaned from this study may be used by drip irrigation engineers to create new emitters that express commercially valuable behaviors, such as lowering activation pressure to reduce pumping power, and reducing material volume (of both the emitter and silicone membrane) to decrease capital costs.

## Limitations of this study

We recognize that the constitutive model of hyper-elasticity would have been more accurate in estimating the deflection of a silicone membrane, and future work will include this phenomena. Hyper-elasticity may explain the slight differences between the experimental flow rates measured when testing at increasing and decreasing pressures. This is because the membrane response may be dependent on strain history and loading rate. We also recognize that modeling the shear deformation of the membrane into the channel as a thick plate, rather than a simply supported thick beam, could improve the accuracy and robustness of our model.

## Conclusions

This paper presents a novel, simplified but high fidelity 2D analytical model of a PC drip emitter that uses a flexible membrane to limit flow. The main goal of this paper was to analyze the coupled fluid-solid interactions within a PC emitter. This work may be extended into creating improved drip emitter designs that provide further value to farmers, by reducing pressure drop (and thus pumping power) and lowering capital cost of the system. Such advancements will facilitate the dissemination of drip irrigation technology to hundreds of millions of poor farmers around the world, who could grow more crops to rise out of poverty while conserving water.

The key insights gleaned from this study are that in order to achieve PC behavior, the resistance to flow in a dripper needs to increase linearly with rising inlet pressure. Manipulation of the flow path area, length, and perimeter are ways to realize this linear increase in resistance. The minimum pressure required to achieve PC behavior is significantly affected by the orifice loss coefficient, which can be influenced by changes in orifice shape and size. In order to maintain PC behavior, the dimensions of channel depth, channel width, channel length, and deflection to the lands can be manipulated, as can the dimensions of the inner land diameter and the diameter, thickness, and material of the membrane. An optimization study on the major geometric parameters within an emitter, in conjunction with the theory presented herein, could be used to determine an optimal geometry and membrane material type to achieve a desired flow rate versus inlet pressure curve.
